# Direct fermentative conversion of poly(ethylene terephthalate) into poly(hydroxyalkanoate) by *Ideonella sakaiensis*

**DOI:** 10.1038/s41598-021-99528-x

**Published:** 2021-10-07

**Authors:** Ryoga Fujiwara, Rikako Sanuki, Hiroharu Ajiro, Toshiaki Fukui, Shosuke Yoshida

**Affiliations:** 1 Graduate School of Biological Science, Nara Institute of Science and Technology, 8916-5, Takayama-cho, Ikoma, Nara 630-0192 Japan; 2 Department of Applied Biology, Kyoto Institute of Technology, Saga Ippongi-cho 1, Ukyo-ku, Kyoto, 616-8354 Japan; 3 Division of Materials Science, Graduate School of Science and Technology, Nara Institute of Science and Technology, 8916-5, Takayama-cho, Ikoma, Nara 630-0192 Japan; 4 School of Life Science and Technology, Tokyo Institute of Technology, 4259 Nagatsuta, Midori-ku, Yokohama, 226-8501 Japan; 5 Division for Research Strategy, Institute for Research Initiatives, Nara Institute of Science and Technology, 8916-5, Takayama-cho, Ikoma, Nara 630-0192 Japan

**Keywords:** Biotechnology, Microbiology, Environmental sciences

## Abstract

Poly(ethylene terephthalate) (PET) is a widely used plastic in bottles and fibers; its waste products pollute the environment owing to its remarkable durability. Recently, *Ideonella sakaiensis* 201-F6 was isolated as a unique bacterium that can degrade and assimilate PET, thus paving the way for the bioremediation and bioconversion of PET waste. We found that this strain harbors a poly(hydroxyalkanoate) (PHA) synthesis gene cluster, which is highly homologous with that of *Cupriavidus necator*, an efficient PHA producer. Cells grown on PET accumulated intracellular PHA at high levels. Collectively, our findings in this study demonstrate that *I. sakaiensis* can mediate the direct conversion of non-biodegradable PET into environment-friendly plastic, providing a new approach for PET recycling.

## Introduction

Petroleum-based plastics are used extensively worldwide. However, owing to their inertness, plastics entering the environment are tough to degrade, and their accumulation has serious negative impacts^1^. Recent reports on microbial degradation of plastics including polyethylene^2^, polystyrene^3^, and poly(ethylene terephthalate) (PET)^4^ highlight these problems, but the number of such reports is limited. Particularly, mechanisms underlying PET biodegradation are best known by analyzing *Ideonella sakaiensis* strain 201-F6^4^. This bacterium uses two unique enzymes, PET hydrolase (PETase) and mono(2-hydroxyethyl) terephthalic acid (MHET) hydrolase (MHETase), to hydrolyze PET into terephthalic acid (TPA) and ethylene glycol (EG) monomers, which are then assimilated. Biodegradable plastics can be developed as more environmentally compatible and sustainable alternatives to common plastics. Poly(hydroxyalkanoate) (PHA), a polyester synthesized by various microorganisms for intracellular carbon and energy storage, is a promising biodegradable plastic, given its excellent biodegradability^5^. A representative PHA-producing strain, *Cupriavidus necator* H16 can produce and accumulate massive quantities of the PHA poly(3-hydroxybutyrate) (PHB), of up to ≈80 w/w% of dry cell weight (DCW, ≈4 g/L culture), using plant oils as the sole carbon source for growth^6^. A critical challenge in microbial fermentation-mediated biodegradable plastic production is lowering the carbon source cost, as it influences the product price^5^. PET is cheap and abundant but deleterious to the environment; thus, the digestion of PET is expected to be a useful carbon source for PHA production. Kenny *et al*.^7^ isolated *Pseudomonas* species, including the GO16 strain, which can accumulate PHAs with monomers comprising more than six carbons (medium chain-length PHAs) from TPA. These strains, when cultured using TPA derived from PET pyrolysis products, accumulated PHA at ≈25 w/w% of DCW (≈1 g/L culture), thereby achieving a PHA production of 0.25 g/L. Tiso *et al*.^8^ modified the GO16 strain for EG and TPA assimilation. They cultured the strain with TPA and EG, which were obtained by complete PET hydrolysis using a thermostable polyester hydrolase, to obtain PHAs at 7 w/w% of DCW (2.3 g/L culture) with a PHA production of 0.15 g/L. Here, we investigated the use of *I. sakaiensis* for directly converting PET into PHA, which does not require prior PET hydrolysis.

## Results and discussion

**Figure 1:**
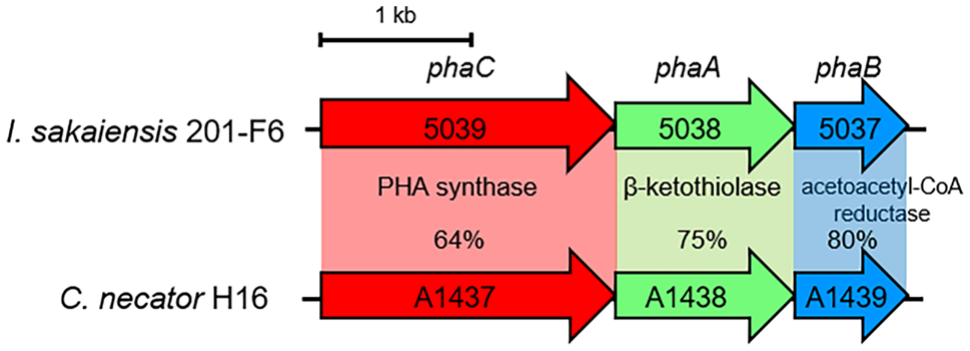
Conservation of poly(hydroxyalkanoate) (PHA) synthesis gene clusters on the genomes of *I. sakaiensis* 201-F6 (GenBank Accession no. NZ_BBYR01000000) and *C. necator* H16 (NZ_CP039287). Open reading frame numbers without locus tag prefixes (ISF6 for *I. sakaiensis*; H16 for *C. necator*) are indicated in the genes. Gene products and amino acid sequence identities are indicated between the genes.

**Figure 2:**
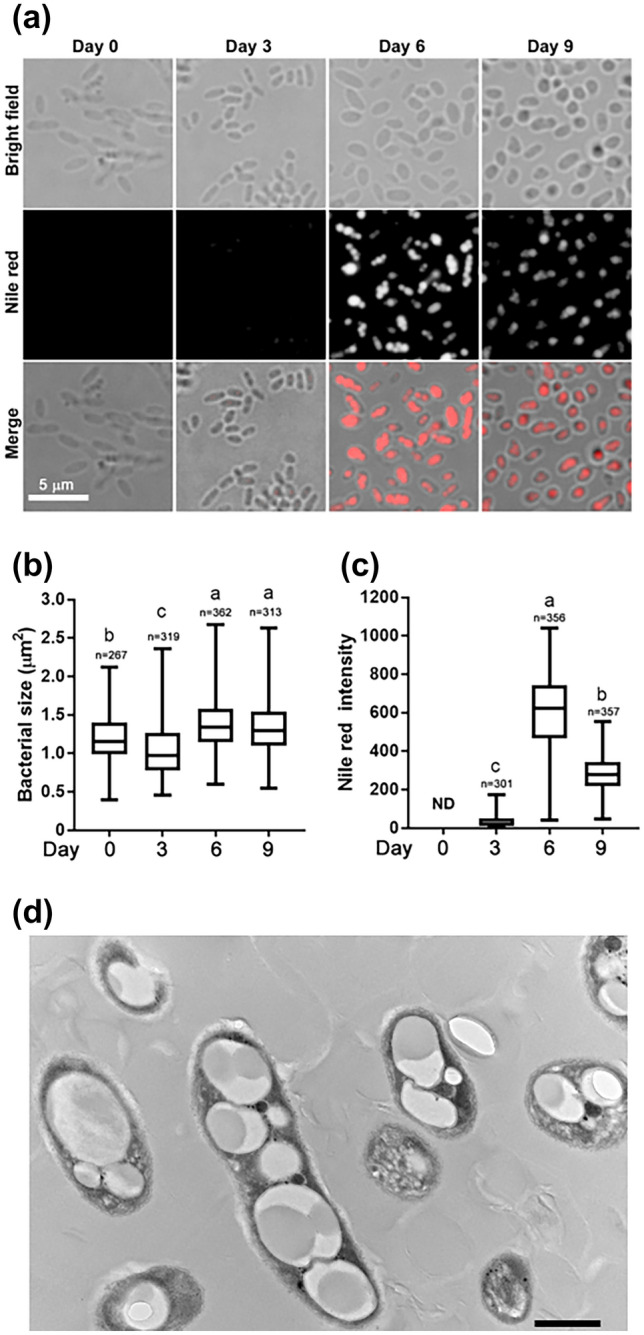
Microscopy of *I. sakaiensis* grown on poly(ethylene terephthalate) (PET). (**a**) Bright field and fluorescence microscopy of Nile red-stained *I. sakaiensis* cells after growth on PET for 0 (pre-cultured cells in 802 nutrient-rich medium for 1 day), 3, 6, and 9 days. Box-and-whisker plots show the distribution of cell areas (**b**) and average fluorescence intensity of Nile red in cells (**c**); median values, 25th to 75th percentiles, and range are shown. n, number of cells or fluorescent dot assemblies. ND, not detected. Different letters above the plots indicate significant differences among groups (Tukey's multiple comparisons test, *P* < 0.0001). (**d**) Transmission electron microscopy image of cells grown on PET granules for 6 days. Scale bar, 600 nm.

**Figure 3:**
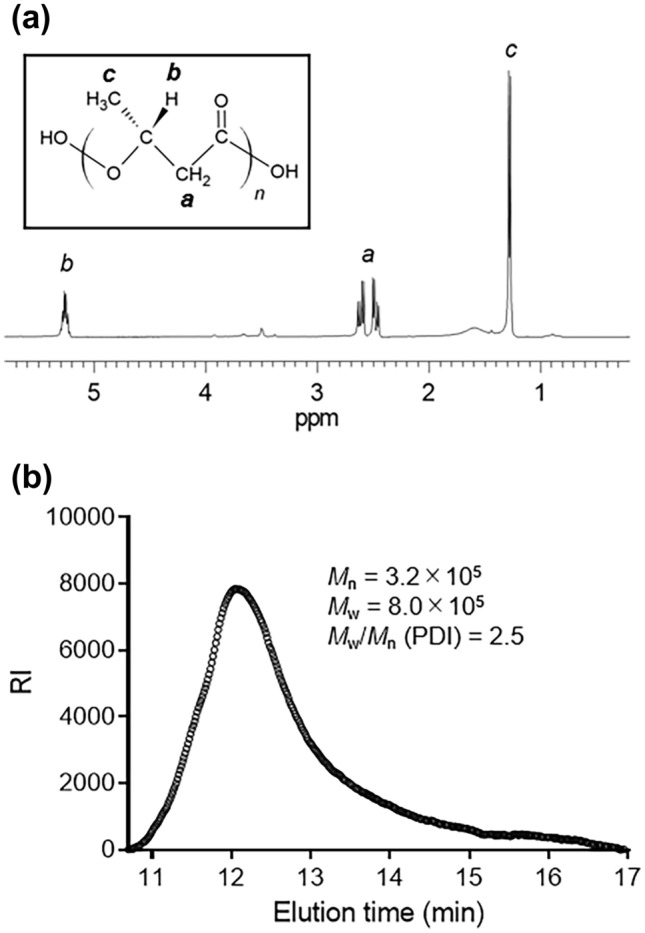
Analyses of chloroform-soluble fraction of *I. sakaiensis* cells grown on poly(ethylene terephthalate) (PET) granules for 6 days. (**a**) Proton nuclear magnetic resonance (^1^H NMR) spectrum. Inlet shows the chemical structure of poly(3-hydroxybutyrate) (PHB). (**b**) Size exclusion chromatography (SEC). *M*_n_, number average molecular weight; *M*_w_, weight average molecular weight; PDI, polydispersity index. Molecular weight values were calculated by comparison with poly(methylmethacrylate) (PMMA) calibration standards.

**Figure 4:**
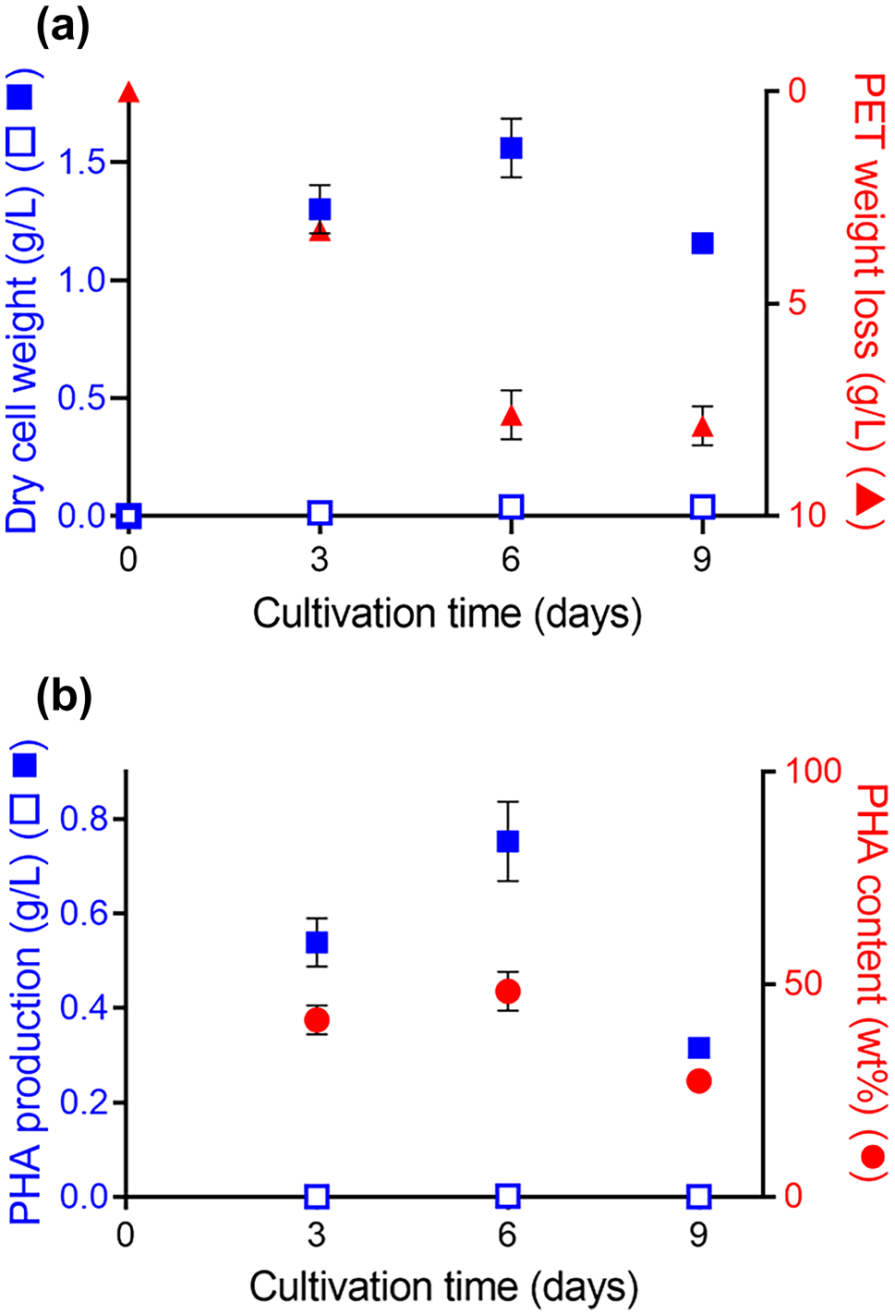
Time course of poly(hydroxyalkanoate) (PHA) production by *I. sakaiensis* grown on poly(ethylene terephthalate) (PET). (**a**) Growth of *I. sakaiensis* on PET. The cells were cultured with and without PET granules (10 g) in YSVO medium for 3, 6, and 9 days at 30 °C. Dry cell weight (DCW; blue closed squares, with PET; blue open squares, without PET) and weight loss of PET granules (red closed triangles) were measured. (**b**) Time course of PHA production. PHA production with and without PET shown as blue closed squares and open squares, respectively. PHA content per DCW shown as red closed circles. Data are also shown in Table 1. Error bars in represent the standard error among three independent biological replicates.

**Figure 5:**
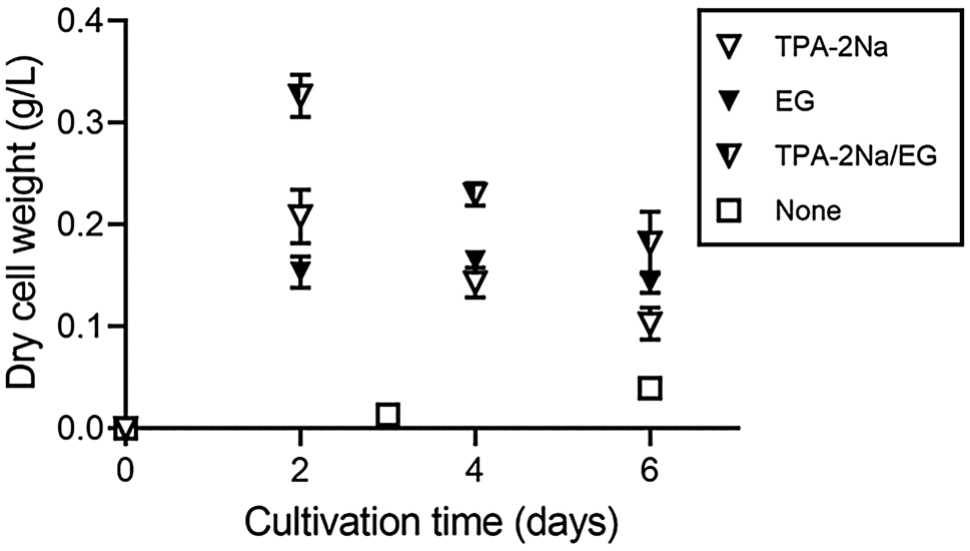
Growth of *I. sakaiensis* on poly(ethylene terephthalate) (PET) monomers. The cells were cultured with or without 0.5 w/v% disodium terephthalate (TPA-2Na), 0.5 w/v% ethylene glycol (EG), and a mixture of the two compounds (TPA-2Na/EG) in YSVO medium at 30 °C, and then their dry cell weight was measured. Error bars in represent the standard error among three independent biological replicates.

We found a gene cluster on the *Ideonella sakaiensis* 201-F6 genome essential for converting acetyl coenzyme A (acetyl-CoA) into PHAs (Fig. 1). The cluster comprises *phaC*, *phaA*, and *phaB* encoding PHA synthase, β-ketothiolase, and acetoacetyl-CoA reductase, respectively. The amino acid sequences of these proteins are highly similar to those of the proteins produced by *C. necator*, indicating that this bacterium is a potential PHB producer. *I. sakaiensis* was cultured on PET granules in a minimal medium (yeast extract-sodium carbonate-vitamins-oyster shell [YSVO] medium) for 3, 6, and 9 days, and stained with Nile red, a lipophilic fluorescent dye. Microscopy revealed that fluorescence intensity in the intracellular regions increased over time with the highest abundance and intensity on day 6 (Fig. 2a,c). This was likely to have affected the bacterial cell area, which increased 1.3-fold between days 3 and 6 (Fig. 2a,b). Transmission electron microscopy (TEM) of the 6-day-cultured cells showed multiple large granules in the cytoplasm (Fig. 2d), which were consistent with those observed using fluorescence microscopy (Fig. 2a). The chloroform-soluble fraction was extracted from the lyophilized *I. sakaiensis* cells cultured on PET and analyzed via proton nuclear magnetic resonance (^1^H NMR) spectroscopy and size exclusion chromatography (SEC) to determine the chemical structure and molecular weight, respectively. The ^1^H NMR spectrum showed typical signals of poly(3-hydroxybutyrate) (PHB), as noted previously^9^ (Fig. 3a). SEC revealed a number-average-molecular weight (*M*_n_) of 3.2 × 10^5^ and a weight-average-molecular weight (*M*_w_) of 8.0 × 10^5^ (Fig. 3b), which are similar to that of PHB produced by *C. necator* (*M*_n_, 4.0 × 10^5^; *M*_w_, 1.0 × 10^6^)^6^. To quantify the PHA content, *I. sakaiensis* was cultured in PET granules-added YSVO medium while monitoring the DCW. We have previously shown that *I. sakaiensis* degrades PET films, exhibited by a significant reduction in their weight^4^ and that it causes an increase in the proportion of the functional (hydroxyl and carboxyl) groups through surface hydrolysis^10^. However, the amount of PET hydrolysates in the culture supernatant was small (0.003% of MHET units of the degraded PET), indicating their rapid uptake into the cell. Therefore, we used the weight loss of PET as another measure of the degradation*. I. sakaiensis* exhibited minimal growth on the culture medium without PET, with the highest DCW of 0.040 g/L, but much higher growth on the medium with PET with DCW of up to 1.6 ± 0.1 g/L, concurrent with a PET weight loss of 7.6 ± 0.6 g/L (Fig. 4a), indicating that PET was a major carbon source for its growth; oyster shell, consisting mostly of calcium carbonate and various trace amounts of minerals^11^, has little to no factors that contribute to cell growth. The PHB content was quantified by performing gas chromatography (GC) of the lyophilized cells after methanolysis. Methyl 3-hydroxybutyrate (3HB) (~ 100 mol%) along with trace amounts of methyl 3-hydroxyvalerate (3HV) were detected among the potential methylated-PHA monomers (Table 1), indicating that the synthesized PHA was nearly a PHB homopolymer. The highest amount of accumulated PHA was observed on day 6, accounting for 48 ± 5 w/w% of the DCW (Fig. 4b, Table 1). The highest PHA production rate was 0.75 ± 0.09 g/L on day 6. Subsequently, the carbon yield of PHA from PET on day 6 was calculated as 9.0 ± 1.5 % (Table 1), indicating that a majority of the carbon in PET was converted into other compounds including carbon dioxide through respiration. We further examined cultivation of *I. sakaiensis* in YSVO medium supplemented with the PET monomer(s); 0.5 w/v% disodium terephthalate (TPA-2Na), 0.5 w/v% EG, or a mixture of both. The DCWs were significantly higher when compared to the culture without carbon source: 0.21 ± 0.03 g/L on TPA-2Na, 0.15 ± 0.02 g/L on EG, and 0.33 ± 0.02 g/L on TPA-2Na/EG for 2 days, and small amounts of PHA (< 5.0 wt%) were detected (Fig. 5 and Table 1). These facts indicated that *I. sakaiensis* was able to incorporate extracellular TPA and EG and then metabolized them to acetyl-CoA and other intermediates. However, considering much high cell growth and PHA accumulation on PET, the cellular ability to assimilate the monomer(s) appeared to be poor. One of explanations for the observations would be better preference of the uptake system of *I. sakaiensis* to PET hydrolytic oligomer products rather than TPA and EG.

**Table 1:** PHA biosynthesis by *Ideonella sakaiensis*.

Carbon source	Cultivation time (days)	DCW (g/L)	PHA (g/L)	PHA content (wt%)	Composition (mol%)		PET weight loss (g/L)	*Carbon yield of PHA from PET (%)
					3HB	3HV		
PET	3	1.3 ± 0.1	0.54 ± 0.05	42 ± 3	~ 100	Trace	3.3 ± 0.1	15 ± 2
	6	1.6 ± 0.1	0.75 ± 0.09	48 ± 5	~ 100	Trace	7.6 ± 0.6	9.0 ± 1.5
	9	1.2 ± 0.0	0.32 ± 0.00	27 ± 1	~ 100	Trace	7.9 ± 0.5	3.6 ± 0.2
TPA-2Na	2	0.21 ± 0.03	0.00059 ± 0.00006	0.28 ± 0.01	100	0		
EG	2	0.15 ± 0.02	0.0073 ± 0.0006	4.9 ± 0.1	100	0		
TPA-2Na/EG	2	0.33 ± 0.02	0.0033 ± 0.0002	1.0 ± 0.0	100	0		
None	3	0.014	0.00039	2.8	100	0		
	6	0.039	0.0019	5.0	100	0		
	9	0.040	0.00022	0.56	100	0		

Error bars represent the standard error among three independent biological replicates.

*DCW* dried cell weight, *EG* ethylene glycol, *ND* not detected, *PET* poly(ethylene terephthalate), *PHA* poly(hydroxyalkanoate), *TPA-2Na* disodium terephthalate, *3HB* 3-hydroxybutyrate, *3HV* 3-hydroxyvalerate.

*Calculated as [(carbon weight of PHA produced with PET—carbon weight of PHA produced without PET)/carbon weight in degraded PET]×100%.

In conclusion, we demonstrated that *I. sakaiensis* could not only metabolize PET, but also produce PHA, and that these two pathways were functionally linked. This information can contribute to the development of new PHA production pathways, which can help reduce plastic pollution and increase the production of inexpensive biodegradable plastics and PET recycling. Compared with previous studies on PHA production from the digestion of PET by *Pseudomonas* GO16^7^ and the modified strain^8^, *I. sakaiensis* produced 3.0- and 5.0-fold more PHA, respectively, from PET. However, scaling up the production levels to an industrial scale remains challenging. The metabolic and fermentation engineering strategies previously applied for *C. necator*, which enable the production of PHA copolymer (> 100 g/L) by high-density fed-batch cultivation under nitrogen limitation^12^, offers a potentially useful approach.

## Methods

### Culture

*I. sakaiensis* (NITE Biological Resource Center, NBRC 110686) was cultured as described previously^4,13^ with several modifications. Briefly, *I. sakaiensis* was pre-cultured in NBRC 802 medium (1.0 w/v% polypeptone, 0.2 w/v% yeast extract, and 0.1 w/v% MgSO_4_) at 30 °C and harvested by centrifugation (5000×*g*, 5 min, 4 °C). The cell pellet was resuspended in YSV medium (0.01 w/v% yeast extract, 0.02 w/v% sodium hydrogen carbonate, 0.1 w/v% ammonium sulfate, 0.01 w/v% calcium carbonate, 0.1 v/v% vitamin mixture [0.25 w/v% thiamine-HCl, 0.005 w/v% biotin, and 0.05 w/v% vitamin B12], and 1 v/v% trace elements [0.1 w/v% FeSO_4_·7H_2_O, 0.1 w/v% MgSO_4_·7H_2_O, 0.01 w/v% CuSO_4_·5H_2_O, 0.01 w/v% MnSO_4_·5H_2_O, and 0.01 w/v% ZnSO_4_·7H_2_O] in 10 mM phosphate buffer; pH 7.4). The suspension was inoculated into a Petri dish (90 mm in diameter) containing 30 mL YSV medium supplemented with ≈ 300 mg of oyster shells as a pH adjusting agent (YSVO medium) in the presence or absence of 10 g (≈ 560 pieces) of PET granules shaped like an elliptic cylinder [2 (minor axis) × 3 (major axis) × 3 (height) mm]; 5.8% crystallinity determined by differential scanning calorimetry (Bell Polyester Products, Inc., Yamaguchi, Japan), 0.5 w/v% of disodium terephthalate (TPA-2Na), EG, or a mixture of 0.5 w/v% TPA-2Na and 0.5 w/v% EG as the carbon source to adjust the absorbance measured at 660 nm to 0.002. The culture dish was placed in a reciprocal shaker set at 50 strokes/min at 30 °C. After filtering the culture fluid through a 5 µm pore size poly-vinylidene difluoride filter (Merck Millipore, Billerica, MA) to remove the small broken pieces of oyster shells, cells were harvested, lyophilized using an FZ-2.5 freeze-dry system (Labconco, Kansas City, MO), and weighed. As a portion of the cells was trapped by the filter, the DCW was corrected using the amounts of protein before and after filtration. Briefly, cells were mixed with an equal volume of 10 w/v% sodium dodecyl sulfate (SDS) and heated at 90 °C for 10 min to lyse the cells and solubilize the cellular protein, and the protein concentration was determined with a DC protein assay kit (BioRad, Hercules, CA) with comparison to a calibration curve using bovine serum albumin. The PET granules were washed with 1 w/v% SDS, distilled water, and then ethanol. After drying in air and a desiccator, the granules were weighed to determine the weight reduction.

### Fluorescence microscopy

PHA that accumulated in the cells was stained with Nile red (Fujifilm Wako Pure Chemical, Osaka, Japan)^14^. A glass slide was coated with 0.01 w/v% poly-l-lysine (Sigma-Aldrich, St. Lois, MO) for cell attachment. The culture suspension (100 µL) was placed on the poly-l-lysine-coated glass slide for 10 min and then aspirated. The cells attached to the glass slide were incubated with a drop of 10 µg/mL Nile red in D-PBS(−) (Nacalai Tesque, Kyoto, Japan) for 30 min, washed with D-PBS(−), mounted with Prolong Diamond antifade medium (Thermo Fisher Scientific, Waltham, MA) for immobilization, and covered with a coverslip. Cells were observed using a fluorescence microscope (BZ-X800, Keyence, Osaka, Japan). The frame of each individual cell was manually identified, and the cell area was calculated using the BZ-X800 Analyzer software (Keyence). PHA formation was visualized using a tetramethylrhodamine isothiocyanate filter set (*λ*_em_ ≈ 554 nm, *λ*_ex_ ≈ 570 nm). The fluorescence intensity of the Nile red-stained dot assembly was calculated on average using the BZ-X800 Analyzer software.

### TEM

The *I. sakaiensis* cells grown on PET granules for 6 days were harvested by centrifugation, fixed with phosphate-buffered 2% glutaraldehyde, and post-fixed with 2% osmium tetraoxide for 3 h on ice. Next, the samples were dehydrated using an ethanol gradient and embedded in epoxy resin. Ultrathin sections of the specimen were stained with uranyl acetate and lead, and subjected to TEM using the H-7600 transmission electron microscope (Hitachi, Tokyo, Japan).

### Polymer extraction and analyses

Samples were prepared as described previously^15^ with several modifications. The lyophilized cells of *I. sakaiensis* were suspended in chloroform with vigorous stirring for 1 day, and the extract was filtered through a polytetrafluoroethylene filter (pore size, 0.2 µm). The filtrate was mixed with twofold volume of 35% methanol:35% ethanol:30% water (v/v/v), and the precipitate was dried *in vacuo* and then dissolved in chloroform. To determine the molecular weight of the polymer, the chloroform-soluble fraction was analyzed using a size exclusion chromatography system equipped with a RI-2031Plus refractive detector (Jasco, Tokyo, Japan) and two in-line TSKgel GMH_HR_-M columns with a TSK-guard column H_HR_-H (Tosoh, Tokyo, Japan) at 40 °C. Chloroform was used as the mobile phase and passed through the column at a flow rate of 1 mL/min. The molecular weight was estimated by comparison with poly(methylmethacrylate) (PMMA) standards (PMMA calibration kit M-M-10, Agilent Technologies, Santa Clara, CA). To analyze the polymer chemical structure, the chloroform-soluble fraction was further purified by precipitation in cold methanol. The precipitate was dissolved in deuterated chloroform (CDCl_3_) and analyzed via proton nuclear magnetic resonance (^1^H NMR) (JNM-ECX400P, Jeol, Tokyo, Japan).

### PHA quantification and composition analysis

The cellular PHA content and composition were determined by GC after direct methanolysis of the dried cells in the presence of 15% sulfuric acid at 100 °C for 140 min, as described previously^16^. The GC analysis was performed using GC-2014 (Shimadzu, Kyoto, Japan) equipped with an InertCap 1 capillary column (30 m × 0.23 mm; GL Sciences, Tokyo, Japan) and a flame ionization detector. PHA composition was further determined by mass spectrometry.
