# Author Correction: Direct fermentative conversion of poly(ethylene terephthalate) into poly(hydroxyalkanoate) by *Ideonella sakaiensis*

**DOI:** 10.1038/s41598-024-66149-z

**Published:** 2024-07-09

**Authors:** Ryoga Fujiwara, Rikako Sanuki, Hiroharu Ajiro, Toshiaki Fukui, Shosuke Yoshida

**Affiliations:** 1 Graduate School of Biological Science, Nara Institute of Science and Technology, 8916‑5, Takayama‑cho, Ikoma, Nara 630‑0192 Japan; 2 Department of Applied Biology, Kyoto Institute of Technology, Saga Ippongi‑cho 1, Ukyo‑ku, Kyoto 616‑8354 Japan; 3 Division of Materials Science, Graduate School of Science and Technology, Nara Institute of Science and Technology, 8916‑5, Takayama‑cho, Ikoma, Nara 630‑0192 Japan; 4 School of Life Science and Technology, Tokyo Institute of Technology, 4259 Nagatsuta, Midori‑ku, Yokohama 226‑8501 Japan; 5 Division for Research Strategy, Institute for Research Initiatives, Nara Institute of Science and Technology, 8916‑5, Takayama‑cho, Ikoma, Nara 630‑0192 Japan

Correction to: *Scientific Reports* 10.1038/s41598-021-99528-x, published online 07 October 2021

The original version of this Article contained errors.

The source of the *Ideonella sakaiensis* strain used in the study was incorrectly attributed.

As a result, in the Methods section, “Culture”

“*Ideonella sakaiensis* was cultured as described previously^4,13^ with several modifications. Briefly, *I. sakaiensis* was pre-cultured in NITE Biological Resource Center (NBRC) 802 medium (1.0 w/v% polypeptone, 0.2 w/v% yeast extract, and 0.1 w/v% MgSO_4_) at 30 °C and harvested by centrifugation (5000×*g*, 5 min, 4 °C).”

now reads:

“*I. sakaiensis* (NITE Biological Resource Center, NBRC 110686) was cultured as described previously^4,13^ with several modifications. Briefly, *I. sakaiensis* was pre-cultured in NBRC 802 medium (1.0 w/v% polypeptone, 0.2 w/v% yeast extract, and 0.1 w/v% MgSO_4_) at 30 °C and harvested by centrifugation (5000×*g*, 5 min, 4 °C).”

Additionally, in the Acknowledgements section

“We are grateful to Prof. Kohei Oda for the kind gift of *I. sakaiensis*. We thank Dr. Tsuyoshi Ando, Yumiko Kawakami, and Mari Nakagawa for research assistance.”

now reads:

“We thank Dr. Tsuyoshi Ando, Yumiko Kawakami, and Mari Nakagawa for research assistance.”

The original Article has been corrected.

